# An 81-Nucleotide Deletion in SARS-CoV-2 ORF7a Identified from Sentinel Surveillance in Arizona (January to March 2020)

**DOI:** 10.1128/JVI.00711-20

**Published:** 2020-07-01

**Authors:** LaRinda A. Holland, Emily A. Kaelin, Rabia Maqsood, Bereket Estifanos, Lily I. Wu, Arvind Varsani, Rolf U. Halden, Brenda G. Hogue, Matthew Scotch, Efrem S. Lim

**Affiliations:** a Center for Fundamental and Applied Microbiomics, Biodesign Institute, Arizona State University, Tempe, Arizona, USA; b School of Life Sciences, Arizona State University, Tempe, Arizona, USA; c Center for Immunotherapy, Vaccines and Virotherapy, Biodesign Institute, Arizona State University, Tempe, Arizona, USA; d Center of Evolution and Medicine, Arizona State University, Tempe, Arizona, USA; e Structural Biology Research Unit, Department of Integrative Biomedical Sciences, University of Cape Town, Observatory, Cape Town, South Africa; f Center for Environmental Health Engineering, Biodesign Institute, Arizona State University, Tempe, Arizona, USA; g One Water One Health Nonprofit Project, Arizona State University Foundation, Tempe, Arizona, USA; h Center for Applied Structural Discovery, Biodesign Institute, Arizona State University, Tempe, Arizona, USA; i College of Health Solutions, Arizona State University, Phoenix, Arizona, USA; Loyola University Chicago

**Keywords:** COVID-19, ORF7a, SARS-CoV-2

## LETTER

On 26 January 2020, the first coronavirus disease 2019 (COVID-19) case was reported in Arizona (third case in the United States) (1). Here, we report on early severe acute respiratory syndrome coronavirus 2 (SARS-CoV-2) sentinel surveillance in Tempe, Arizona. Genomic characterization identified an isolate encoding a 27-amino-acid in-frame deletion in accessory protein ORF7a, the ortholog of SARS-CoV immune antagonist ORF7a/X4.

**FIG 1:**
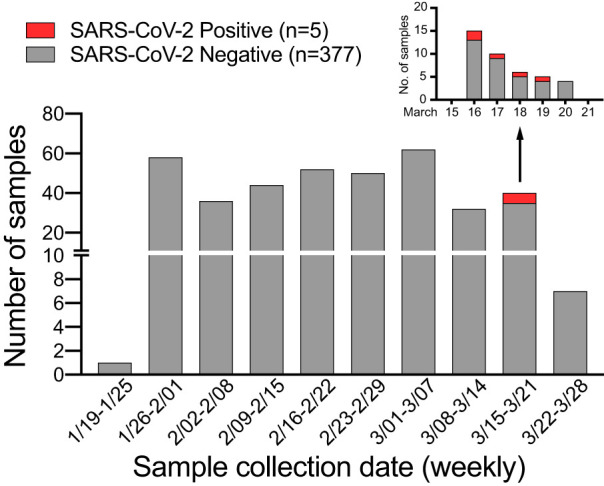
SARS-CoV-2 surveillance in Tempe, AZ, from January to March 2020. Shown is the weekly distribution of NP specimens collected by ASU Health Services and tested for SARS-CoV-2 by qRT-PCR assays. The inset shows SARS-CoV-2-positive NP specimens collected from the week of 15 to 21 March 2020.

In anticipation of COVID-19 spreading in Arizona, we initiated a surveillance effort for the local emergence of SARS-CoV-2 starting 24 January 2020. We leveraged an ongoing influenza surveillance project at Arizona State University (ASU) Health Services in Tempe, Arizona. Individuals presenting with respiratory symptoms (ILI) were tested for influenza A and B viruses (Alere BinaxNOW). Subsequently, we tested influenza-negative nasopharyngeal (NP) swabs for SARS-CoV-2. We extracted total nucleic acid using the bioMérieux eMAG automated platform and performed real-time quantitative reverse transcription-PCR (qRT-PCR) assays specific for SARS-CoV-2 N and E genes (2, 3). Out of 382 NP swabs collected from 24 January to 25 March 2020, we detected SARS-CoV-2 in 5 swabs from 16 to 19 March (Fig. 1). This corresponds to a prevalence of 1.31%. Given the estimated 1- to 14-day incubation period for COVID-19, the spike in cases might be related to university spring break holiday travel (8 to 15 March), as previously seen in other outbreaks (4, 5).

**FIG 2:**
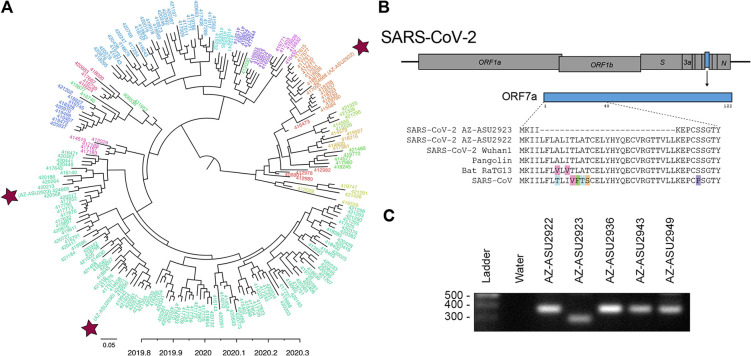
Evolutionary and genomic characterization of SARS-CoV-2 genomes. (A) Bayesian maximum clade credibility (MCC) polar phylogeny of 222 full-length SARS-CoV-2 genomes. The 3 new genomes reported in this study are indicated by red stars. Sequences were aligned in Geneious prime (version 2020.0.3) using the MAFFT v7.450 plugin, and the 5′ and 3′ untranslated regions (<300 nt each) were trimmed. We initiated two independent runs of 500M sampling every 50K steps and used Tracer v1.7.1 (18) to check for convergence and that all effective sample size (ESS) values for our statistics were >200, LogCombiner (7) to combine the models with a 10% burn-in, and TreeAnnotator (7) to produce an MCC tree. We used FigTree, v1.4.4 (19), to edit the tree and color the tips based on lineages (6) and pangolin (20) to identify the lineages of our 3 new sequences based on the established nomenclature (6). The nomenclature consists of two main lineages, A and B, and includes sublineages (A.1, B.2, etc.) up to four levels deep (e.g., A.1.1, B.2.1, etc.) (6). For visualization purposes, we grouped all viruses that were not directly assigned to A or B into their first sublineage level and colored tip labels by lineage. B.1 lineage, AZ-ASU2923 and AZ-ASU2936; A.1 lineage, AZ-ASU2922. (B) ORF7a amino acid alignment of SARS-CoV-2 and related genomes. GenBank and GISAID accession numbers were the following: SARS-CoV-2 AZ-ASU2922, MT339039 and EPI_ISL_424668; SARS-CoV-2 AZ-ASU2923, MT339040 and EPI_ISL_424669; SARS-CoV-2 AZ-ASU2936, MT339041 and EPI_ISL_424671; SARS-CoV-2 Wuhan1, MN908947.3; Pangolin, EPI_ISL_410721; Bat-RaTG13, MN996532.1; and SARS-CoV, AY278741.1. The 81-nt (27-amino-acid) deletion observed in SARS-CoV-2 AZ_ASU2923 ORF7a was not present in the 6,290 SARS-CoV-2 sequences available from GISAID as of 12 April 2020. (C) We performed molecular validation by RT-PCR on specimen total nucleic acid extracts with primers flanking the ORF7a N terminus region. The expected size of amplicons with an intact ORF7a region is 377 bp, and the expected size of an amplicon with the NGS-identified 81-nt deletion is 296 bp. Primers were SARS2-27144F, 5′-ACAGACCATTCCAGTAGCAGTG-3′, and SARS2-27520r, 5′-TGCCCTCGTATGTTCCAGAAG-3′.

To understand the evolutionary relationships and characterize the SARS-CoV-2 genomes, we performed next-generation sequencing (NGS; Illumina NextSeq, 2×76) directly on specimen RNA, thereby avoiding cell culture passage and potentially associated mutations. This generated an NGS data set of 20.7 to 22.7 million paired-end reads per sample. We mapped quality-filtered reads to a reference SARS-CoV-2 genome (MN908947) using BBMap (version 39.64) to generate three full-length genomes: AZ-ASU2922 (376× coverage), AZ-ASU2923 (50×), and AZ-ASU2936 (879×) (Geneious prime, version 2020.0.5). We aligned a total of 222 SARS-CoV-2 genome sequences comprising at least 5 representative sequences from phylogenetic lineages defined by Rambaut et al. (6). We performed phylogenetic reconstruction with BEAST (version 1.10.4; strict molecular clock, HKY + Γ nucleotide substitution, exponential growth for coalescent model) (7–10). The ASU sequences were phylogenetically distinct, indicating independent transmissions (Fig. 2A).

Like SARS-CoV, the SARS-CoV-2 genome carries multiple open reading frames in the 3′ region. We found that the SARS-CoV-2 AZ-ASU2923 genome has an 81-nucleotide (nt) deletion in the ORF7a gene, resulting in a 27-amino-acid in-frame deletion (Fig. 2B). The SARS-CoV ORF7a ortholog is a viral antagonist of host restriction factor BST-2/Tetherin and induces apoptosis (11–14). Based on the SARS-CoV ORF7a structure (15), the 27-amino-acid deletion in SARS-CoV-2 ORF7a maps to the putative signal peptide (partial) and first two beta strands. To validate the deletion, we performed RT-PCR using primers spanning the region and verified the amplicons by Sanger sequencing (Fig. 2C). Similar deletions in SARS-CoV-2 genomes are emerging, notably in the ORF8 gene (16), that may reduce virus fitness (17). Hence, further experiments are needed to determine the functional consequences of the ORF7a deletion.

Collectively, although global NGS efforts indicate that SARS-CoV-2 genomes are relatively stable, dynamic mutations can be selected in symptomatic individuals.
